# The influence of the fetal leg position on the outcome in vaginally intended deliveries out of breech presentation at term – A FRABAT prospective cohort study

**DOI:** 10.1371/journal.pone.0225546

**Published:** 2019-12-02

**Authors:** Lukas Jennewein, Roman Allert, Charlotte J. Möllmann, Bettina Paul, Ulrikke Kielland-Kaisen, Florian J. Raimann, Dörthe Brüggmann, Frank Louwen

**Affiliations:** 1 FRABAT FRAnkfurt Breech At Term Study Group; Department of Gynecology and Obstetrics, School of Medicine, Goethe-University, Theodor-Stern-Kai 7, Frankfurt, Germany; 2 Department of Anesthesiology, Intensive Care Medicine and Pain Therapy, University Hospital Frankfurt, Frankfurt am Main, Germany; Ospedale dei Bambini Vittore Buzzi, ITALY

## Abstract

**Introduction:**

Vaginal delivery out of a breech presentation in pregnancies at term are being re-implemented into clinical practice. Still, recommendations regarding exclusion criteria leading to caesarean sections are based on expert opinions, not on evidence-based guidelines. The difference in perinatal outcome and course of delivery in births with babies in frank breech position and babies in incomplete or complete breech presentation never has been investigated in a large patient cohort.

**Objective:**

To compare perinatal outcome of vaginally intended breech deliveries between births out of frank breech position and incomplete/complete breech presentation.

**Design:**

Prospective cohort study.

**Sample:**

884 women at term with a singleton in frank breech presentation (FB) and 284 women with incomplete or complete breech presentation (CB) intending vaginal birth between January 2004 and December 2018.

**Methods:**

Maternal and fetal outcome was compared between groups using Pearson’s Chi Square test. Birth duration parameters were analysed using logistic regression.

**Results:**

There were no differences in cesarean section rates (FB: 25.1%, CB 22.2%, p = 0.317). Short-term fetal morbidity did not differ between groups (FB: 2.5%, CB: 2.8%, p = 0.761). In vaginal deliveries the necessity to perform manual assistance was significantly more frequent in deliveries of infants in CB (FB: 39.9%, CB: 51.6%, p = 0.0013). Cord loops (FB: 10.1%, CB: 18.0%, p = 0.0004) and cesarean sections necessary because of cord prolapses (FB: 1.4%, CB 8.1%, p = 0.005) were significantly more often in deliveries with babies in CB.

**Conclusion:**

This study provides evidence, that perinatal morbidity is not associated with the fetal leg posture in vaginally intended breech deliveries. The higher risk for the need of manual assistance during vaginal birth in deliveries of babies out of complete or incomplete breech presentation suggests that obstetrical departments re-implementing the vaginal breech in their repertoire might start with births of babies out of frank breech presentation.

## Introduction

Fetal breech presentation at term occurs in 3–4% of pregnancies [1]. Hence, vaginal breech delivery should be included in every obstetrician’s repertoire but clinical expertise and experience have constantly declined over the last decades. Many providers in high-income countries recommend a planned cesarean section as the safest delivery mode for breech infants. This trend is problematic since growing perinatal morbidity and mortality parallel rising cesarean section numbers [2–6]. Excessive postpartum bleeding, placenta percreta and uterine rupture are potentially life threatening complications for mother and child and best prevented by reducing their most important risk factor–a previous cesarean section. As an alternative to a cesarean section, eligible patients should be offered an external cephalic version to promote delivery out of vertex presentation [7]. In patient counseling, providers should clarify that success rates of this procedure are reported to be below 50% [8]. Hence, a delivery out of breech presentation still needs to be addressed thoroughly in patients counseling.

National guidelines differ in their recommendations on breech management. Hence, it is necessary to provide more evidence on delivery outcomes in order to unify international standards [9]. For instance the British guideline emphasizes the safety of the vaginal approach when a baby presents as breech at term and certain prerequisites are met [7]. Conditions such as a skilled obstetrical team, an adequate pelvis with an intertubarous distance greater than 11 cm or an upright birth position have proven their value in order to secure patient safety [10,11]. However, many existing precautions and exclusion criteria for a vaginal birth approach are vague, intuitively chosen or opinion based. For example, vaginal delivery of infants out of incomplete or a complete breech presentation (one or both legs downwards leading together with the fetal coccyx) is deemed to be less complicated compared to a delivery out of frank breech presentation (both legs up, fetal coccyx leading) [12]. To our knowledge overall data on association of adverse perinatal outcome and the posture of the fetal legs in vaginal breech births is scarce. One study investigating this particular matter was performed in a small cohort of 266 patients. Here, Krause et al. reported a higher rate of short term neonatal morbidity in deliveries out of incomplete and complete breech presentation [13].

To promote the safety of vaginal breech deliveries, facilitate clinical decision making in patients counseling and promote unified guidelines in the future, more consistent evidence is needed on the role of the fetal leg posture in vaginally intended breech deliveries at term. Hence, this study aimed at comparing (1) the maternal and fetal outcome and (2) the mode of delivery between patients who intended a vaginal delivery of a baby in frank breech against complete and incomplete breech.

## Materials and methods

### Patient cohort and patient selection

**Fig 1:**
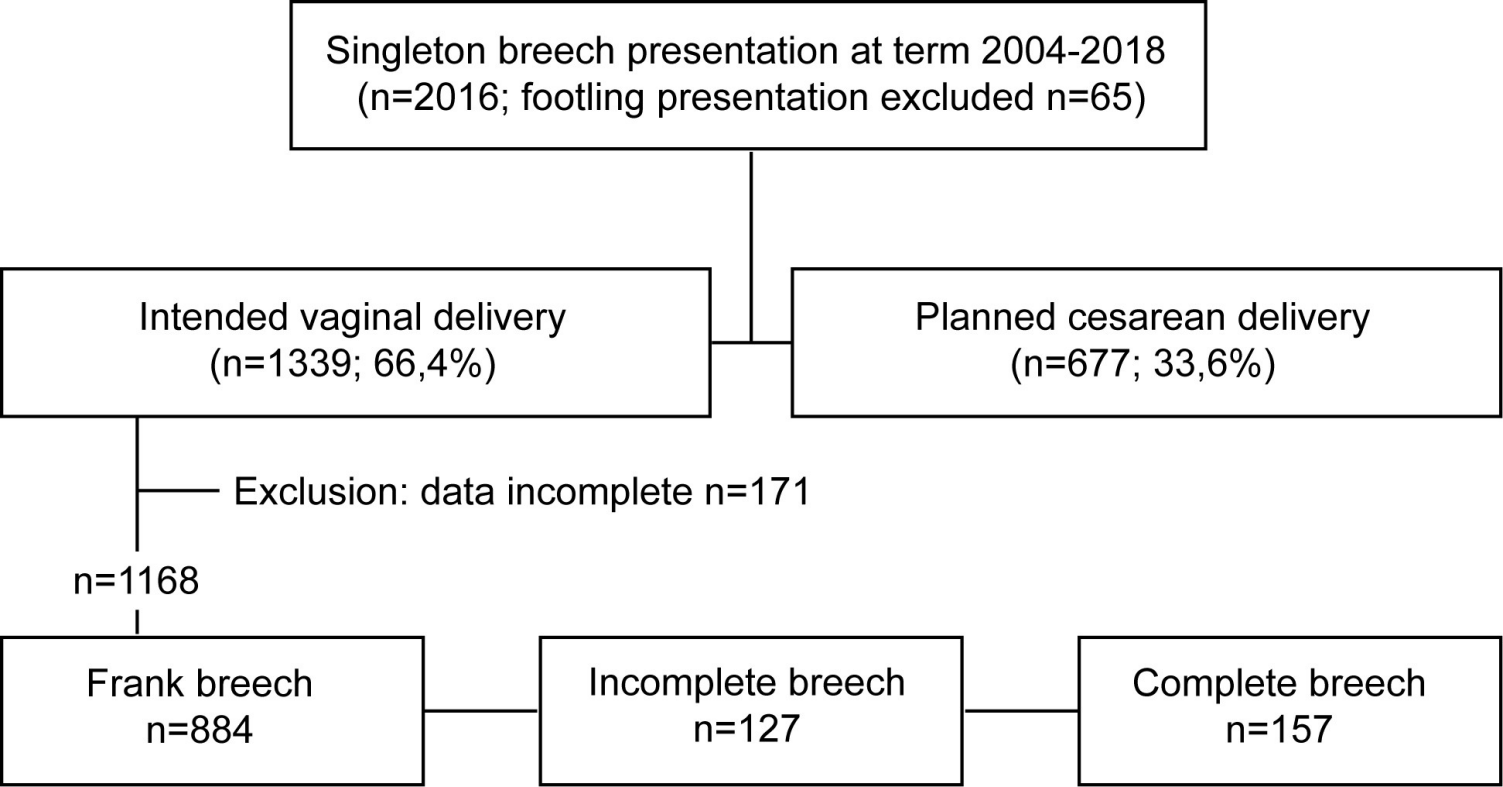
Flow chart of study cohort.

From January 2004 until December 2018, we performed a prospective case-controlled study on all women presenting with a fetus in breech presentation at term (>37 weeks of pregnancy) at the Goethe University Hospital Frankfurt. The university hospital’s ethics committee gave consent (420/11). Because the patient information was gathered after patients dismissal from the hospital and no patient was treated otherwise than within standard clinical care, the ethics committee waved a patient's consent. Data was analyzed anonymously. Of 2016 women presenting with a fetus in breech presentation at term, 1168 were included (Week of gestation > 36+6, vaginal intended breech delivery). Reasons for exclusion were the following: planned cesarean delivery, infant’s birth weight of below 2.5 kg or footling presentation (Fig 1).

‘Perinatalerhebung Hessen’, a state database was used for data collection. Data was completed using the hospitals patient management system. All data was gathered after discharge of the patients.

The counseling process, as well as patient selection and procedures at our center has been previously described [11,14].

**Fig 2:**
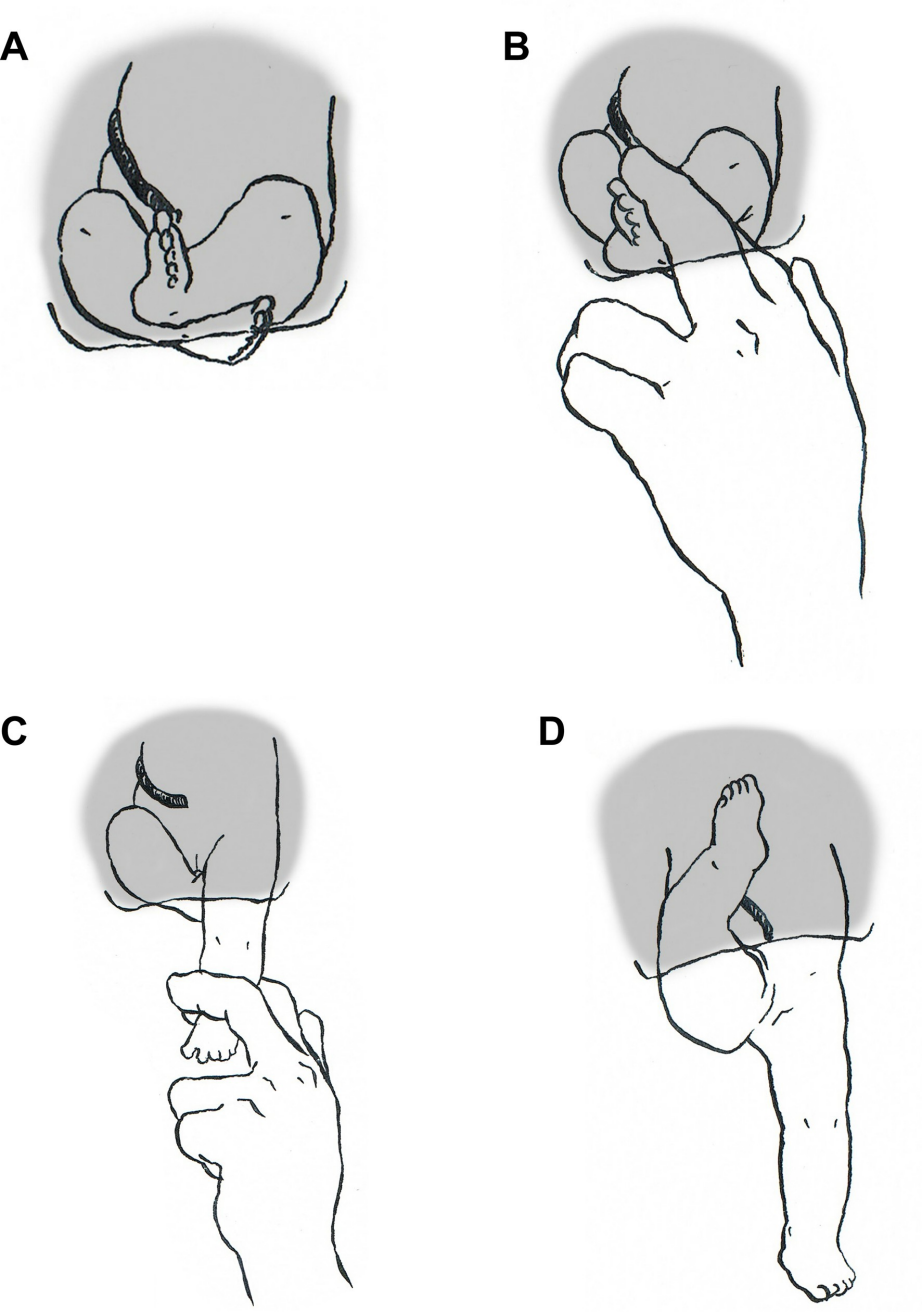
Cartoon of release maneuver in order to loosen a wedged leg in a complete breech presentation. A) baby’s position in the pelvis when the mother lies on the back (only while clinical examination the mother will be placed on the back during birth in our center) B) The examiner grabs the lower foot with two fingers. C) The examiner pulls the leg out. D) Position of the baby after the maneuver. Figure published with the permission of the artist, Chloe Dolic.

The preferred maternal position during delivery (stage II) in all spontaneous and manually assisted vaginal deliveries was in upright position (e.g. on knees and arms). In some cases, the obstetrician in charge changed birth position individually. In incomplete and complete breech presentation, the leading leg can wedge and cause (i) pain and (ii) arrest of delivery. In our center, it is common practice to offer peridural anesthesia because of this known phenomenon. If a wedged leg inhibits birth progression (which might be the case in complete breech presentation), it is standard procedure in our center to release the leg manually without pulling the baby. The maneuver is shown in Fig 2. Afterwards, spontaneous course of delivery is possible again. This maneuver was not counted as a manual assistance because in all cases, leg manipulation and actual delivery with or without manual assistance did not happen in immediate timely order.

### Statistical analyses

Group differences were tested using Pearson’s χ^2^ test and Fishers exact test. Compared groups consisted of unequal sample sizes. To additionally demonstrate statistical accuracy, all tests were performed after equalizing sample sizes through random selection of patients within the larger group (frank breech group) [14,15]. There were no different results after performing those analyzes if not otherwise indicated.

To analyze birth duration, mean and standard deviation were estimated. Since normal distribution did not apply (birth duration in all vaginal deliveries: Shapiro-Wilk-W-Test W = 0.887) and sample sizes were unequal, non-parametric Wilcoxon test was performed to detect differences.

Statistical analyzes were conducted using JMP software (SAS Institute, Cary, USA).

## Results

1168 pregnant women at term with a fetus in breech presentation decided to intend vaginal delivery at our center. 884 patients presented with a baby in frank breech presentation (coccyx leading, both legs upwards; FB). In 284 cases, one or both legs were leading in addition to the coccyx (incomplete and complete breech presentation; CB). Age, BMI and duration of pregnancy was equally distributed between groups. In the FB group, there were more primiparous women (61.2% in comparison to the CB group (51.1%)). Fetal leg position was not associated with maternal preconditions or uterus abnormalities (Table 1). The cesarean section rate after onset of labor was not significantly different (FB: 25.1%, CB: 22.2%, p = 0.317) (Table 1).

**Table 1:** General characteristics vaginal intended deliveries FB vs. CB.

Characteristic	Frank breech N = 884	Incomplete and complete breech N = 284	P Value
**Age (mean, SD)**	32.2 (±4.2)	32.2 (±4.9)	0.979
**BMI (mean, SD)**	23.1 (±3.9)	23.0 (±4.0)	0.360
**Duration of pregnancy in days** **(mean, SD)**	279 (±8.2)	278 (±8.4)	0.097
**Parity** (n, %)			0.009
1	541, 61.2%	145, 51,1%	
2	218, 24.7%	92, 32.4%	
> 2	125, 14.1%	47, 16.6%	
**Fetal birth weight (gram; mean, SD)**	3346 (412, ±14)	3380 (418, ±25)	0.280
**Maternal preconditions count**	140,15.8%	35, 12.3%	0.149
**Abnormal uterus conditions (myomas, previous cesarean)**	37, 4.2%	18, 6.3%	0.136
**Delivery mode**			0.003
Spontaneous vaginal birth	402, 45.5%	107, 37.7%	
Manually assisted birth	260, 29.4%	114, 40.1%	
Cesarean section	222, 25.1%	63, 22.2%	0.317

We analyzed differences in cesarean section indications in all abdominal deliveries after initial vaginal approach (FB cesarean group n = 222, CB cesarean group n = 63). There were no significant differences in rates of birth arrest or non-reassuring heart rate pattern (Table 2). Notably, umbilical cord prolapse as an indication to perform a cesarean section happened significantly more often in the CB group (FB: 1.4%, CB: 8.1%, p = 0.005, Table 2).

**Table 2:** Cesarean section indications all Cesarean deliveries FB vs. CB.

Characteristic	Frank breech N = 222	Incomplete and complete breech N = 63	P Value
**Mother’s wish count**	7 (3.2%)	3 (4.8%)	0.540
**Arrest in Stage I**	79 (35.4%)	17 (27.0%)	0.202
**Arrest in Stage II**	65 (29.2%)	18 (28.6%)	0.913
**Non-reassuring fetal heart rate pattern**	77 (34.7)	17 (27.0)	0.251
**Umbilical cord prolapse**	3 (1.4%)	5 (7.9%)	0.005
**Suspected infection (chorioamnionitis)**	7 (3.2%)	0 (0.0%)	0.154

Fetal outcome was evaluated using several parameters (fetal arterial pH, 5’ APGAR score, admission to the neonatal intensive care unit (NICU), birth trauma, intubation, neurological deficit, neonatal death). Each parameter on its own did not show a significant difference when all vaginal intended deliveries were compared between FB and CB group. A modified PREMODA score was used [14,16] to refine our analysis. There was no significant difference in neonatal morbidity possibly related to mode of delivery (after exclusion of cases of chorioamnionitis, perinatal infection and congenital fetal malformations/diseases) between FB group (2.5%) and CB group (2.8%) with a p value of 0.761 (Table 3). Nuchal cords or umbilical cord loops around other parts of the baby’s body as findings during or after delivery were found significantly more often in births out of CB (18%) in comparison to deliveries out of FB (10.1%, p = 0.0004).

**Table 3:** Fetal outcome parameters vaginal intended deliveries FB vs. CB.

Characteristic	Frank breech N = 884	*Incomplete and complete breech* *N = 284*	P Value
**PREMODA Score count**	49 (5.5%)	15 (5.3%)	0.866
**PREMODA Score rel. to mode of delivery** *	19 (2.2%)**	8 (2.8%)***	0.515
**pHa < 7.0**	5 (0,6%)	2 (0.7%)	0.796
**5’ APGAR < 4**	5 (0.6%)	1 (0.4%)	0.662
**NICU > 4 days**	44 (5.3%)	15 (5.0%)	0.839
**Birth trauma** (e.g. clavicular or humerus fracture)	8 (0.9%)	3 (1.1%)	0.818
**Intubation > 24h**	9 (1.0%)	2 (0.7%)	0.634
**Neurological deficit** (intracranial bleeding, seizures)	6 (0.7%)	2 (0.7%)	0.964
**Neonatal death**	1 (0.1%)	0 (0.0%)	0.571
**Infection**	40 (4.5%)	7 (2.5%)	0.124
**Umbilical cord loop**	89 (10.1%)	51 (18.0%)	0.0004

* The following cases were excluded (from PROMODA Score to PREMODA rel. to delivery mode, leading cause mentioned): FB-group: 21 cases of newborn infection, one case of aspiration and pneumothorax, two cases of spontaneous intracerebral bleeding, one case of death 7 days after birth of a newborn with Potter syndrome, two case of arrhythmia, one case of bradycardia because of a triple umbilical cord loop around the neck (emergency caesarean section), one case of pulmonary stenosis, one case of myoclonism, CB-group: four cases of newborn infection, one case of meconium ileus, one case of congenital heart disease, one case of congenital hypothyroidism, one case of glucose-6-phosphate-dehydrogenase deficiency.

** 2 cases of forceps delivery of the following head with perinatal asphyxia, 2 cases of plexus paralysis with perinatal asphyxia, 1 case of clavicular fracture without perinatal asphyxia, 1 case of cephalic fracture and perinatal asphyxia where a forceps had to be performed on the following head without neurologic deficiencies at discharge. 1 case of plexus paralysis without perinatal asphyxia, 1 case of isolated humerus fracture after assisted delivery of arms without asphyxia, 1 case of plexus paralysis and severe problems adapting after birth, 1 case of severe problem adapting after birth with a pulmonary hypertension, 1 case of perinatal asphyxia with pneumothorax (with spontaneous recovery) and renal bleeding, 8 cases of perinatal asphyxia

*** 1 case of forceps delivery of the following head with perinatal asphyxia, 2 cases of uncomplicated clavicular fracture without asphyxia, 5 cases of perinatal asphyxia

All infants were discharged in good general condition.

When all vaginal deliveries (vaginal frank breech = vFB, vaginal complete or incomplete breech = vCB) were analyzed separately, a significantly higher amount of manual assistance was noticed (vFB 39.9%, CB 51.6%, p = 0.0013). Assistance of head delivery was significantly more often necessary in the vCB group (FB: 33.5%, CB 35.7%). The Louwen maneuver (help with delivery of arms while the mother is on all fours [11]) was performed significantly more frequently in vCB cases (FB: 23.7%, CB: 29.9%, p = 0.0069). Need for peridural anesthesia as well as maternal birth injuries were not significantly different between both groups (Table 4). Of note, duration of second stage was significantly longer in deliveries of babies in frank breech presentation (FB = 61 minutes, CB = 47 minutes, p = 0.013, Table 4). Neonatal morbidity possibly related to the mode of delivery was not different between groups of vaginal deliveries (vFB 2.4%, CB 3.2%, p = 0.544, Table 4).

**Table 4:** Fetal outcome parameters vaginal deliveries FB vs. CB.

Characteristic	Frank breech N = 662	Incomplete and complete breech N = 221	P Value
**Manual assistance**	260 (39.3%)	114 (51.6%)	0.0013
Assistance with head delivery [%]	222 (33.5%)	101 (45.7%)	0.001
Assistance with arm delivery (Louwen maneuver) [%]	157 (23.7%)	66 (29.9%)	0.0069
**Peridural anesthesia**	336 (50.8%)	104 (47.1%)	0.341
**Duration of birth in minutes (**mean, SD**)** *	399 (±308)	363 (±302)	0.179
Duration Stage I*	337 (±280)	313 (±280)	0.286
Duration Stage II*	61 (±72)	47 (±63)	0.013
**Perineal injury**	328 (50.0%)	106 (48.0%)	0.684
**Perineal injury III-IV°**	12 (1.8%)	5 (2.3%)	0.674
**PREMODA Score**	28 (4.2%)	12 (5.4%)	0.458
**PREMODA Score rel. to mode of delivery**	15 (2.3%)	7 (3.2%)	0.457

* data incomplete in 278 cases and thus excluded from analysis

In our collective, parity is not equally distributed (see Table 1). Because it is known that primiparous women experience longer deliveries and cesarean section rate is higher compared to multiparous women, we performed a separate analysis only with primiparous women. Within this analysis, all abovementioned results were comparable except the birth duration means of vaginal deliveries of second stage. Here, no significant difference was detected between vaginal deliveries of babies out of frank breech (83 minutes) versus babies out of incomplete or complete breech presentation (74 minutes, p = 0.247) (Table 5).

**Table 5:** Delivery outcome parameters of all deliveries of primiparous women FB vs. CB.

Characteristic	Frank breech N = 541	Incomplete and complete breech N = 145	P Value
**Spontaneous birth** count (%)	195 (36.0%)	40 (27.6%)	0.024
**Manual assistance**	158 (29.2%)	59 (40.7%)	0.008
**Cesarean section**	188 (34.8%)	46 (31.7%)	0.495
**PREMODA related to mode of delivery**	15 (2.8%)	6 (4.1%)	0.397
**Vaginal Deliveries**	**N = 353**	**N = 99**	
**Manual assistance**	158 (44.8%)	59 (59.6%)	0.009
**PDA**	224 (63.5%)	61 (61.6%)	0.738
**Perineal injury**	191 (54.1%)	56 (56.6%)	0.664
**Perineal injury III-IV°**	10 (2.8%)	3 (3.0%)	0.917
**Duration of birth in minutes (**mean, SD**)** *	514 (±314)	471 (±364)	0.120
**Duration of stage I** *	528 (±290)	392 (±341)	0.084
**Duration of stage II** *	83 (±83)	74 (±80)	0.247

* Data of 140 cases missing, thereby excluded from analysis.

**Fig 3:**
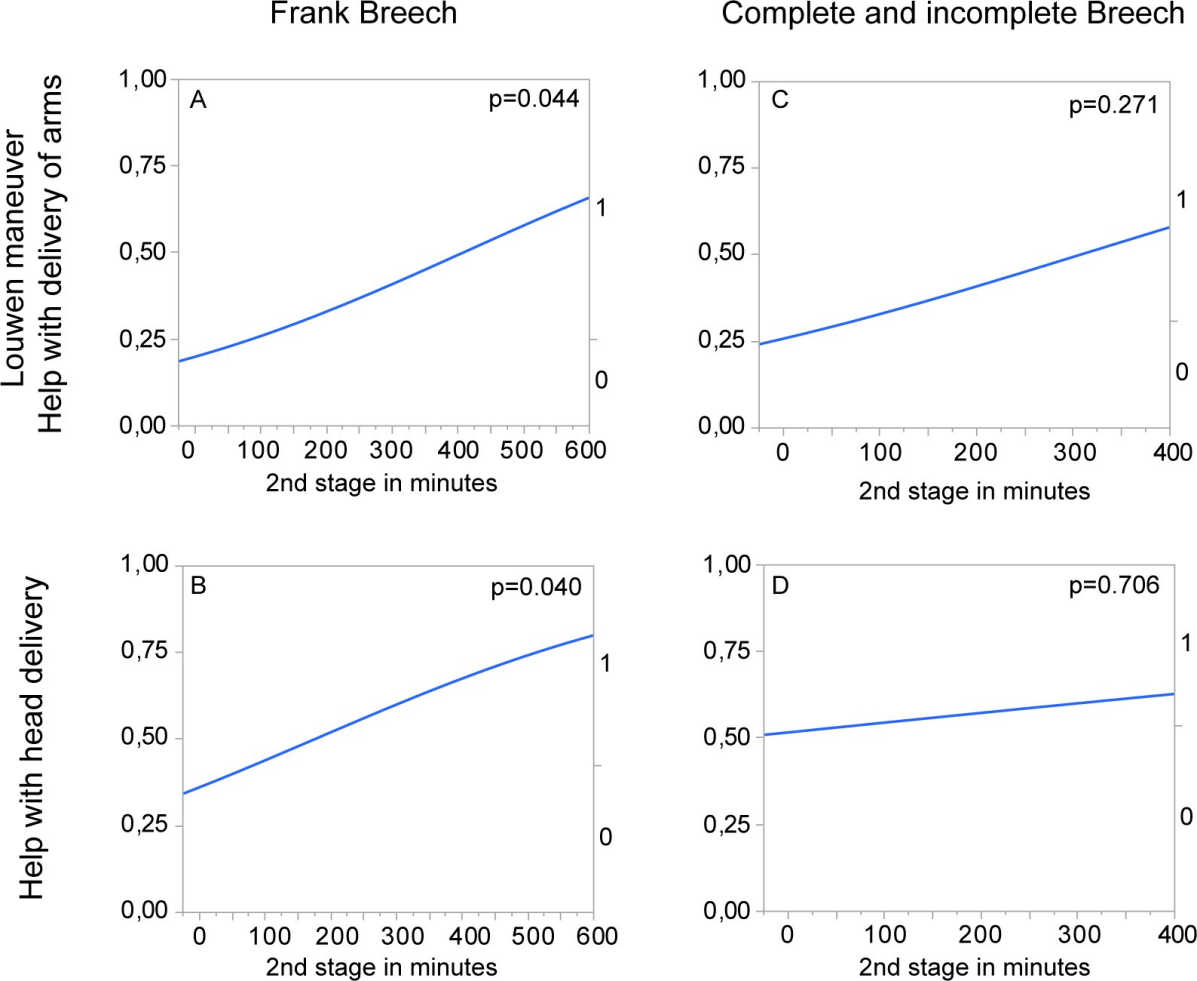
Logistic regression of second stage with manual assistance in primipara Logistic regression analysis of all vaginal deliveries of primiparous women. Display with inverse y axis in order to show graphical tendency of likelihood of manual assistance in relation to the duration of second stage. A) n = 241, logistic regression of duration of second stage to help with delivery of arms (Louwen maneuver) in frank breech vaginal deliveries, Odds ratio per minute: 1.0034, Chi^2^ over-all model test p = 0.0443 B) n = 241, logistic regression of duration of second stage to help with head delivery in frank breech vaginal deliveries, Odds ratio per minute: 1.0033, Chi^2^ over-all model test p = 0.0402 C) n = 71, logistic regression of duration of second stage to help with arm delivery in complete and incomplete breech vaginal deliveries; Odds ratio per minute: 1.0035, Chi^2^ over-all model test p = 0.2707 D) n = 71, logistic regression of duration of second stage to help with head delivery in complete and incomplete breech vaginal deliveries; Odds ratio per minute: 1.0011, Chi^2^ over-all model test p = 0.7059.

Since the fetal leg posture correlated with duration of second stage, we aimed at assessing the association between second stage duration and need for manual assistance. Because parity influences birth duration, we only included primiparous women in this analysis. In vFB deliveries the duration of second stage showed a significant positive correlation with (i) likelihood for manual assistance of delivery of arms (Fig 3A, p = 0.044) and (ii) likelihood for manual assistance of delivery of the head (Fig 3B, p = 0.040). In the respective correlation of duration of second stage and manual assistance in vCB deliveries, no significant correlation was found (Fig 3C and 3D).

## Discussion

Intended vaginal breech deliveries should be re-implemented into the obstetricians routine since national guidelines—once again—recommend the vaginal approach as a safe option when the patient collective is well selected [7,17]. Frequently, clinicians still have reservations against vaginal breech birth, which are based on expert opinions and low-evidence level information from textbooks, leading to insufficient patient information during clinical counseling. This can be directly translated into an unnecessary high cesarean section rate [18,19]. It is important to supply obstetrician’s knowledge with evidence-based information in order to develop well-educated informed consent when patients seek counseling for term breech birth. Because no reliable data is available regarding the impact of the fetal leg position (frank breech, incomplete or complete breech) on perinatal outcome, we extracted data from a large cohort comparing the fetal short-time outcome, the cesarean section rate and the maternal outcome of deliveries of babies out of frank breech presentation vs. incomplete/complete breech presentation.

In our analysis, we saw that the posture of legs has only little impact on perinatal outcome. Neonatal morbidity rates, maternal injuries as well as cesarean section rates did not differ between FB and CB group in intended vaginal deliveries (Table 1). When cesarean section indications were compared, umbilical cord prolapse as a reason for an abdominal delivery occurred significantly more often in CB deliveries than in FB deliveries (Table 2). Also, our data showed that nuchal cords are more frequently found in CB presentations (see Table 3). Breech presentation is reported to be a risk factor for cord prolapses [20–22]. We here can provide additional evidence that the risk for cord prolapse is increased specifically in cases with incomplete or complete breech.

The need to perform manual assistance for the delivery of arms and head during birth was significantly higher in vaginal deliveries of babies in incomplete or complete breech presentation. This can be explained by a shorter second stage of labor in CB cases (Table 4, Fig 3). The baby’s body needs time to rotate in the second stage, enabling the shoulders to enter the pelvic entrance crossways. A shorter second stage could be caused by early manipulation in complete breech cases. When wedging of the leading leg in the birth canal occurs, the lower leg is pulled down and out (Fig 2). The mother’s pain and possible arrest in second stage can be overcome by this maneuver. Besides the baby not having enough time to rotate adequately in the pelvis triggered by early manipulation, the obligatory rotations in the pelvic cavity can also be complicated by the presence of a leg in addition to the fetal coccyx leading to more obstetrical interventions and ultimately to assisted arm and head delivery.

In our first analysis, a significantly longer second stage of labor was detected in deliveries out of a frank breech presentation. This might be explained by a greater percentage of spontaneous births where no accelerating manipulations were performed. Also, the duration of birth is strongly influenced by parity [23,24]. Because parity is not equally distributed in the FB and CB groups, our first analysis might be confounded. When only primiparous women were selected and delivery durations were compared no significant differences were detectable (see Table 5). Duration of second stage correlated positively with need for manual assistance in frank breech deliveries but not in complete or incomplete breech presentation deliveries (Fig 3). This might be due to the abovementioned leg manipulations in complete breech deliveries, shortening the second stage and leading to a favorable position of the baby within the birth canal.

When counseling women expecting a breech baby it is important to explain all options and outcome eventualities. This includes the likelihood of manual assistance and umbilical cord prolapse during birth, which is–according to this study—influenced by the fetal leg posture. Nevertheless, it should be addressed that the fetal leg positioning might change between counseling and labor. Based on our data, this situation would not constitute an indication for a planned cesarean section since perinatal morbidity rates were not significantly different in any analysis between different leg posture variations in our cohort.

Morbidity rates in our study population were increased compared to cephalic deliveries. For instance, nationwide German official reports on perinatal morbidity in 2017 present the following numbers for short-term delivery outcomes of vertex fetuses: postpartum admission to the NICU: 3.14% (vs. 8.4% in our study), 5’ APGAR scores below 4: 0.25% (vs.0.5% in our study), pH below 7.0: 0.25% (vs. 0.6% in our study) (S1 Table) [25]. This observation was not surprising since this well-known fact is mentioned in the British Green top guideline [7]. More importantly, studies with great sample sizes report–in contrast to the Hannah trial [26]–that long term outcome in intended vaginal breech deliveries is comparable to cephalic deliveries even though higher short term morbidity rates exist [16,27].

After the immense decline of vaginal breech deliveries in response to the Term Breech Trial [26] that identified cesarean sections as the favored approach, now the practice of “vaginal breech deliveries” is being re-implemented into the obstetrician’s repertoire. Many authors criticize the trial by Hannah et al. and its methodology [16,19,28,29]. Numerous substantial studies have been published since 2001 and the vaginal approach has been re-instated into national guidelines and committee opinions [7,17]. Until now, little evidence-based information has been gathered to specify and train the new generation of obstetricians who have to learn vaginal breech deliveries. To support obstetrical institutions in re-instating the vaginal option for women at term with a fetus in breech presentation, it might be helpful to know odds and specific characteristics of different breech positions during birth.

In that sense, this study’s results could greatly impact clinical management of breech presentation. Since fetal leg position did not impact the perinatal outcome or the mode of delivery it should not be considered as a decision criterion in clinical counseling. Independent of the fetal leg position, patients should be encouraged to attempt a vaginal breech delivery. It is a strength of this study that our research question has never been addressed prospectively before. A weakness of our data might be that compared groups are not matched in age, BMI and parity, possibly leading to bias. Since only parity was significantly differently distributed between groups (Table 1), we included an analysis of only primiparous women. Because these data was gathered only at one single center, representability might be limited. Multi-center controlled trials should deliver even more reliable data in order to impact international guidelines.

In this trial, we provide evidence that a complete or incomplete breech presentation does not lead to an increased rate of fetal morbidity but requires a skilled obstetric care because manual assistance is more frequently required. Also, the type of breech presentation did not alter maternal outcome or mode of delivery. Obstetrical centers re-implementing a vaginal breech regiment might start with deliveries out of frank breech presentation to gather experience since more spontaneous deliveries can be expected.

## Supporting information

S1 Table Overall descriptive statistics of vaginally intended deliveries. (DOCX)
