# Supplementary material for: The influence of the fetal leg position on the outcome in vaginally intended deliveries out of breech presentation at term – A FRABAT prospective cohort study
Source: PLoS One. 2019 Dec 2;14(12):e0225546. doi: 10.1371/journal.pone.0225546 (PMC6886779; doi:10.1371/journal.pone.0225546)
Supplement: S1 Table — (DOCX) [file pone.0225546.s001.docx]

| **Characteristic** | **Vaginal intended deliveries**  **N=1168** |
| --- | --- |
| **Age (mean, st.dev.)** | 32.0 (± 4.4) |
| **BMI (mean, st.dev.)** | 23.1 (± 4.0) |
| **Duration of pregnancy in days**  **(mean, st. dev.)** | 279.1 (± 8.3) |
| **Parity** (n, %) |  |
| 1 | 686 (58.7 %) |
| 2 | 310 (26.5 %) |
| > 2 | 172 (14.7 %) |
| **Fetal birth weight (gramm; mean, st.dev.)** | 3354 (± 413) |
| **Delivery mode** |  |
| Spontaneous vaginal birth | 509 (43.6 %) |
| Manually assisted birth | 374 (32.0 %) |
| Cesarean section | 285 (24.4 %) |
| **PDA** | 618 (52.9 %) |
| **Birth position in vaginal deliveries**  **(n=883)** |  |
| Upright position | 720 (81.5 %) |
| Doral position | 163 (18.5 %) |
| **PROMDA potentially related to delivery mode** | 27 (2,3 %) |
| **Umbilical pH (arterial) < 7.0** | 7 (0.6%) |
| **5’ APGAR Score < 4** | 6 (0.5%) |
| **NICU Admission** | 98 (8.4%) |
